# Topical *N*-Acetylcysteine Accelerates Wound Healing *in Vitro* and *in Vivo* via the PKC/Stat3 Pathway

**DOI:** 10.3390/ijms15057563

**Published:** 2014-05-02

**Authors:** Min-Ling Tsai, Hui-Pei Huang, Jeng-Dong Hsu, Yung-Rung Lai, Yu-Ping Hsiao, Fung-Jou Lu, Horng-Rong Chang

**Affiliations:** 1 Institute of Medicine, Chung Shan Medical University, Taichung 40201, Taiwan; E-Mails: cshd034@csh.org.tw (M.-L.T.); missyuping@gmail.com (Y.-P.H.); fjlu@csmu.edu.tw (F.-J.L.); 2 Department of Pharmacy, Chung Shan Medical University Hospital, Taichung 40201, Taiwan; E-Mail: cshd050@csh.org.tw; 3 Institute of Biochemistry and Biotechnology, Chung Shan Medical University, Taichung 40201, Taiwan; E-Mail: hhpei@csmu.edu.tw; 4 Department of Pathology, Chung Shan Medical University Hospital, Taichung 40201, Taiwan; E-Mail: dongdong@csmu.edu.tw; 5 Department of Dermatology, Chung Shan Medical University Hospital, Taichung 40201, Taiwan; 6 Division of Nephrology, Department of Internal Medicine, Chung Shan Medical University Hospital, Taichung 40201, Taiwan

**Keywords:** *N*-acetylcysteine, wound healing, glutathione, MMP-1, PKC, Stat3

## Abstract

*N*-Acetylcysteine (Nac) is an antioxidant administered in both oral and injectable forms. In this study, we used Nac topically to treat burn wounds *in vitro* and *in vivo* to investigate mechanisms of action. *In vitro*, we monitored glutathione levels, cell proliferation, migration, scratch-wound healing activities and the epithelialization-related proteins, matrixmetalloproteinase-1 (MMP-1) and proteins involved in regulating the expression of MMP-1 in CCD-966SK cells treated with Nac. Various Nac concentrations (0.1, 0.5, and 1.0 mM) increased glutathione levels, cell viability, scratch-wound healing activities and migration abilities of CCD-966SK cells in a dose-dependent manner. The MMP-1 expression of CCD-966SK cells treated with 1.0 mM Nac for 24 h was significantly increased. Levels of phosphatidylinositol 3-kinase (PI3K), protein kinase C (PKC), janus kinase 1 (Jak1), signal transducer and activator of transcription 3 (Stat3), c-Fos and Jun, but not extracellular signal-regulated protein kinases 1 and 2 (Erk1/2), were also significantly increased in a dose-dependent manner compared to the controls. In addition, Nac induced collagenous expression of MMP-1 via the PKC/Stat3 signaling pathway. *In vivo*, a burn wound healing rat model was applied to assess the stimulation activity and histopathological effects of Nac, with 3.0% Nac-treated wounds being found to show better characteristics on re-epithelialization. Our results demonstrated that Nac can potentially promote wound healing activity, and may be a promising drug to accelerate burn wound healing.

## 1. Introduction

Wound healing involves a complex series of interactions between different cell types, cytokine mediators, and the extracellular matrix (ECM). In general, there are three or four major stages of wound healing: inflammation, proliferation, matrix deposition and remodeling [1,2]. Thermal trauma is an acute wound, where substantial areas of skin can be damaged, often without the possibility of skin regeneration. Burns and scalds can sometimes result in rapid, extensive, deep wounds that cannot be successfully treated with common techniques, and can lead to death [3]. Burn injuries incur a significant cost to health care systems worldwide. Approximately 1.2 million people sustain burn injuries each year and about 100,000 of these cases require hospitalization. Annual estimates show that more than US$18 billion is spent on specialized care of patients with a major burn injury in America [4]. Therefore, burn wounds represent a significant burden to patients, health care professionals, and the health care system.

*N*-Acetylcysteine (Nac), an antioxidant sulfhydryl substance, is a precursor in the formation of glutathione (GSH) in the body. During the process of wound healing, various inflammatory cells such as neutrophils, macrophages, endothelial cells and fibroblasts produce reactive oxygen species (ROS) [5]. GSH is an antioxidant that prevents damage to important cellular components caused by ROS [6], and nuclear GSH is a key regulator of epigenetic events that may be critical in the regulation of cell proliferation, a vital process in wound healing [7]. In addition, Nac has been used clinically to treat a variety of conditions including acetaminophen toxicity, acquired immune deficiency syndrome, cystic fibrosis, chronic obstructive pulmonary disease, diabetes [8], hearing loss [9], perioperative atrial fibrillation [10], acute cholestasis-induced renal failure [11], and acute smoke inhalation injury [12]. The administration of oral antioxidants, vitamin C, vitamin E, and Nac, has shown beneficial effects in the treatment of burn patients, as evidenced by a reduced incidence of wound infections and the shortening of healing time [13]. Nac, in both its oral and injectable forms, is a convenient, safe, and inexpensive medicine for burn wounds. Deniz *et al.* demonstrated that Nac administration via an oral or intraperitoneal route was beneficial for severe burns in a rat comb-burn model [14]. Demir *et al.* reported that intraperitoneally or orally administered Nac improved wound healing in irradiated rats [15]. Topical *N*-acetylcysteine 8% eye drops have also been shown to be effective in the early treatment of experimental alkali corneal burns [16]. However, the potential benefits and mechanisms of topical Nac administration are unclear in the treatment of burn wounds. If topical Nac therapy proves to be a successful therapeutic modality for the treatment of wounds, it may significantly decrease the cost of treatment and thus substantially broaden the scope of patients eligible for treatment.

It has also been reported that collagenase may promote endothelial cell and keratinocyte migration, thereby stimulating angiogenesis and epithelialization in the wound healing process [17]. The ECM consists of structural fibrillar collagens that are remodeled or degraded by matrix metalloproteinases (MMPs), and predominantly MMP-1, which degrades the interstitial collagen and basement membrane proteins to facilitate epithelialization and neovascularization [2,18,19].

Signal transducer and activator of transcription 3 (Stat3) is a cytoplasmic protein that conveys signals to the nucleus, and has been widely shown to play critical roles in biological activities including cell proliferation, migration and survival [20,21]. Furthermore, the protein kinase C (PKC) signaling pathway is a major regulator of cellular function and has been implicated in pathologies involving ECM remodeling. Key roles of PKC in the induction of MMP-1 via Stat3 and extracellular signal-regulated kinases (Erk)-dependent c-Fos induction have also been reported [22]. However, it is unknown whether Nac can promote collagenous expressions by stimulating the activation of PKC-related proteins such as phosphatidylinositol 3′-kinase (PI3K) in the PKC pathway, which allows for the next step of collagen synthesis to proceed.

To test this hypothesis, we designed *in vitro* and *in vivo* experiments to examine how Nac influences the repair process, including cell proliferation, migration, and scratch wound healing. Furthermore, we examined the dose responsive effects of Nac on GSH and MMP-1 activities, phosphatidylinositol 3-kinase (PI3K), PKC, janus kinase 1 (Jak1), Stat3, c-Fos, Jun and Erk1/2 expressions, and burn wound healing in rats.

## 2. Results and Discussion

### 2.1. Results

#### 2.1.1. *N*-Acetylcysteine (Nac) Enhanced Proliferation of CCD-966SK Cells

**Figure 1.**
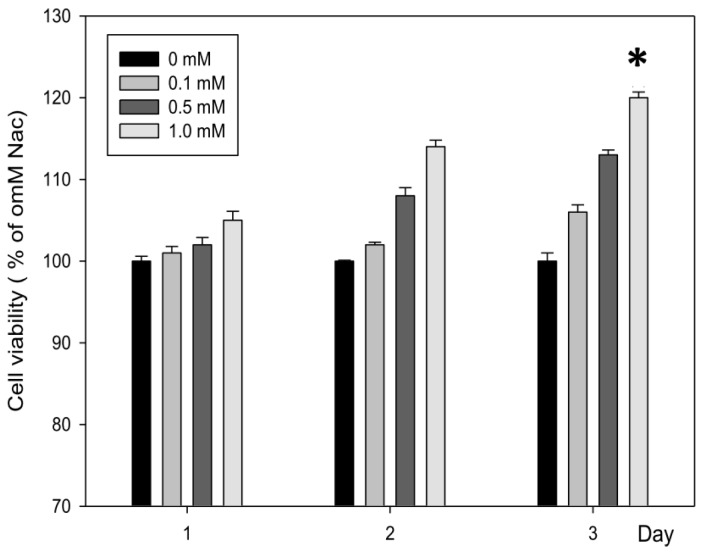
Effect of *N*-Acetylcysteine (Nac) on the viability of CCD-966SK cells. CCD-966SK cells (1 × 10^4^ cells/mL) were treated with various concentrations (0–1.0 mM) of Nac for 1, 2, and 3 days. The viability of the cells was determined by MTT assay. The number of cells that survived was directly proportional to formazan, which was measured spectrophotometrically at 563 nm. Values are expressed as the mean ± SD of three independent experiments. * *p* < 0.01 compared with the untreated controls.

According to the results of cell viability from the MTT (3-(4,5-Dimethylthiazol-2-yl)-2,5-diphenyltetrazolium bromide) assay as shown in Figure 1, there was a proliferation of CCD-966SK cells treated with Nac at different concentrations (0.1–1.0 mM) for 1, 2, and 3 days. Nac showed dose- and time-dependent growth effects on the CCD-966SK cells. A concentration of 1.0 mM Nac led to a significant increase in the number of viable cells at day 3. Thus, in the following experiments, doses below 1.0 mM for 3 days of Nac treatment were used.

#### 2.1.2. Nac Increased the glutathione (GSH) Level of the CCD-966SK Cells

**Figure 2.**
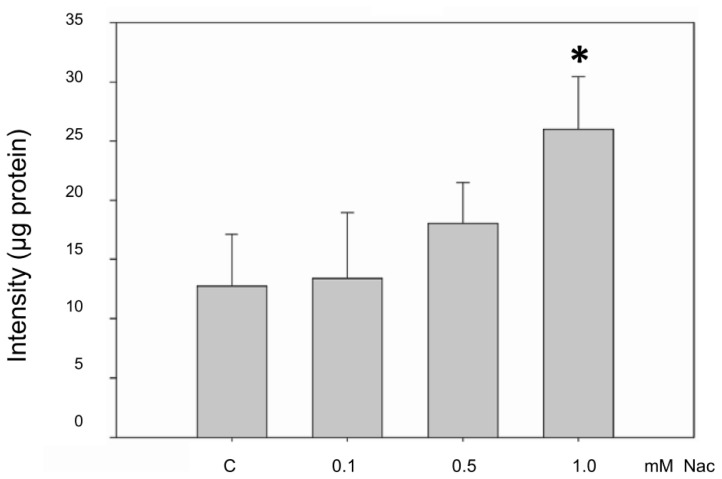
Glutathione (GSH) analysis of the CCD-966SK cells treated with Nac. CCD-966SK cells (1 × 10^5^ cells/mL) were treated with various concentrations (0–1.0 mM) of Nac for 1 day. GSH was measured at *Ex*/*Em* 350/420 nm using fluorescence spectroscopy. The increase of absorbance at 420 nm was expressed as the mean ± SD of three independent experiments. * *p* < 0.01 compared with the untreated controls.

Nac has been reported to stimulate GSH synthesis, enhance GSH-*S*-transferase activity, promote detoxification, and act as a scavenger of free radicals in the liver [23]. Thus, we evaluated the effects of Nac on GSH in CCD-966SK cells. The contents of GSH in the CCD-966SK cells obtained from the control and cells treated with 0.1 mM of Nac were similar, and the GSH level was slightly increased in the cells exposed to 0.5 mM Nac. Nevertheless, the GSH level in the cells treated with a higher concentration of Nac (1.0 mM) significantly increased to approximately double that of the controls (Figure 2).

#### 2.1.3. Wound Healing Ability of the CCD-966SK Cells Treated with Nac

**Figure 3.**
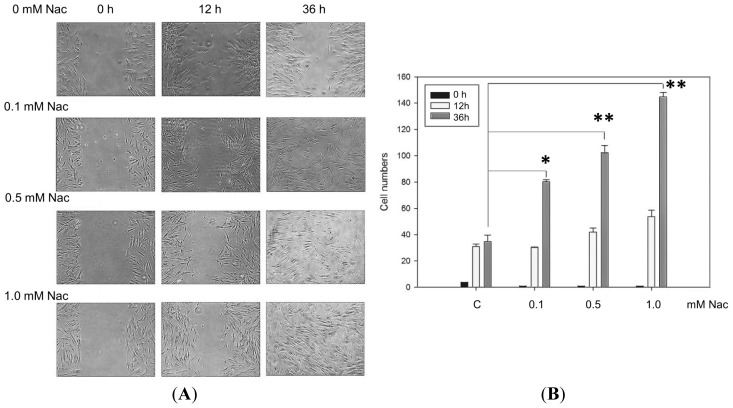
Wound healing analysis of the CCD-966SK cells treated with Nac. (**A**) The treated cells were cultured in a six-well plate after monolayer wounding with a yellow P200 pipette tip. The healing ability was observed using phase contrast microscopy on an inverted microscope after 12 and 36 h; (**B**) Quantitative assessment of the average number of cells in the denuded zone is expressed as the mean ± SD of three independent experiments. * *p* < 0.01; ** *p* < 0.001 compared with the untreated controls.

*In vitro* wound healing assays have commonly been applied to measure cell migration, cell proliferation and wound closure in response to stimulation with specific agents. Treatment of CCD-966SK cells with various concentrations of Nac induced cell migration away from the edge of the cell monolayer and toward the wounded area. Figure 3A demonstrates that the rates of wound closure in the control and treated cells were similar 12 h after wounding. However, the cells treated with Nac (0.1, 0.5, and 1.0 mM) showed a higher rate of wound closure than the controls 36 h after wounding. The cells treated with 1.0 mM Nac had the greatest healing effect among these cells. The activities of wound healing (number of cells in denuded areas) were not different among the 4 groups of Nac after 12 h, however 36 h later, the cells treated with 0.1 mM (80.3 ± 1.5 cells), 0.5 mM (102.3 ± 5.5 cells), and 1.0 mM (144.7 ± 3.5 cells) of Nac all displayed significantly increased healing ability compared to the untreated controls (Figure 3B).

#### 2.1.4. Nac Promoted the Migration of CCD-966SK Cells

**Figure 4.**
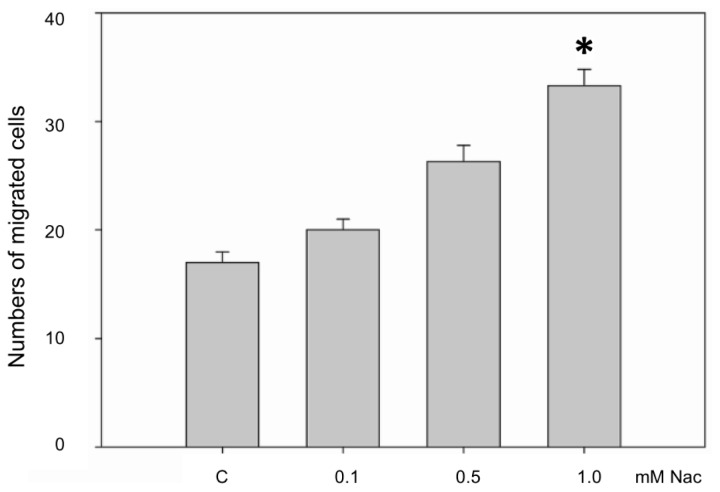
Effect of Nac on migration of CCD-966SK cells. CCD-966SK cells were treated with various concentrations (0–1.0 mM) of Nac for one day. Cell migration was measured in a Transwell assay for 12 h with Millicell (Millipore, Bedford, MA, USA). The migration ability of CCD-966SK cells was quantified by counting the number of cells that invaded the underside of the porous polycarbonate membrane under microscopy, and is represented as the average of three experiments, mean ± SD. * *p* < 0.01 compared with the untreated controls.

Cell migration is a highly integrated, multi-step process that plays an important role in wound healing. We tested the migratory ability of CCD-966SK cells treated with various concentrations of Nac using a Boyden chamber assay. As seen in Figure 4, Nac significantly induced the migration of CCD-966SK cells in a dose-dependent manner, whereas the migration abilities of CCD-966SK cells treated with 0.1 and 0.5 mM Nac did not differ from the controls. However, the migration activity of CCD-966SK cells treated with 1.0 mM Nac for one day was significantly higher than that of the controls.

#### 2.1.5. Nac Promoted Matrixmetalloproteinase-1 (MMP-1) Expression in CCD-966SK Cells through Phosphatidylinositol 3-Kinase (PI3K) and Signal Transducer and Activator of Transcription 3 (Stat3) Signaling

**Figure 5.**
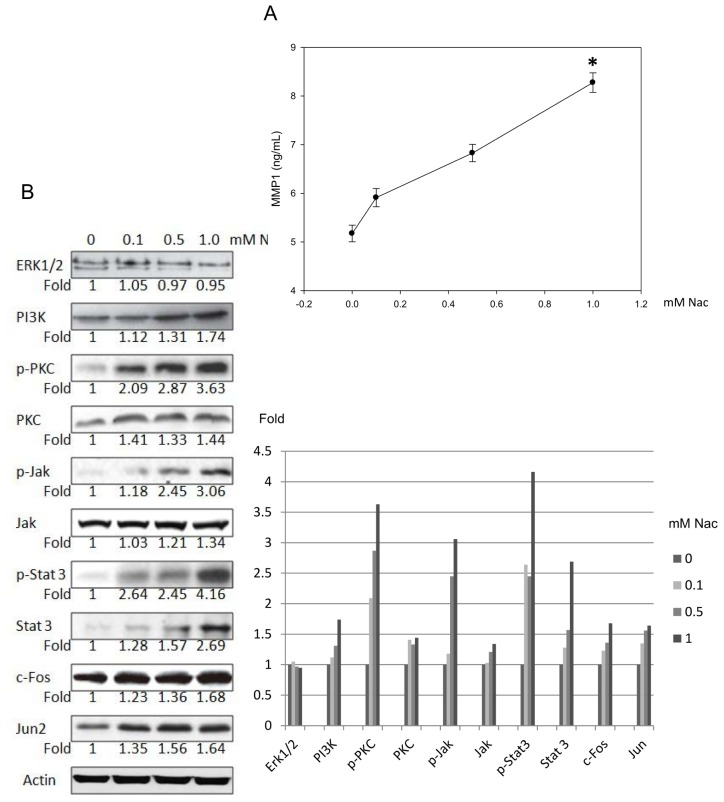

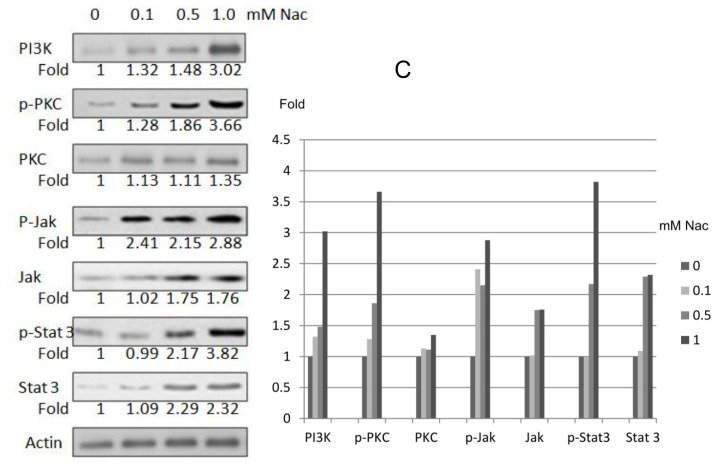
The expressions of Matrixmetalloproteinase-1 (MMP-1), extracellular signal-regulated protein kinases 1 and 2 (Erk1/2), phosphatidylinositol 3-kinase (PI3K), phospho-protein kinase C (p-PKC), PKC, phospho-Janus kinase 1 (p-Jak1), Jak1, phospho-Signal transducer and activator of transcription 3 (*P*-Stat 3), Stat 3, c-Fos and Jun induced by Nac in CCD-966SK cells. (**A**) After treatment with various concentrations (0–1.0 mM) of Nac for 1 day, MMP-1 expressions were quantified by enzyme-linked immunosorbent assay (ELISA); (**B**) After treatment with various concentrations (0–1.0 mM) of Nac for 1 day, the protein levels of Erk1/2, PI3K, p-PKC, PKC, p-Jak1, Jak1, p-Stat 3, Stat 3, c-Fos, and Jun involved in regulating the expression of MMP-1 were analyzed and quantified by Western blotting; (**C**) After scratch wound healing assay, post-wounded CCD-996SK cells were collected and the protein levels of PI3K, p-PKC, PKC, p-Jak, Jak, p-Stat 3 and Stat 3 were analyzed and quantified by Western blotting. The data are presented as the mean ± SD of at least three independent experiments. * *p* < 0.01 compared with the untreated controls.

Enzyme-linked immunosorbent assays (ELISA) were used to clarify the MMP-1 expression in the CCD-966SK cells induced by Nac. As seen in Figure 5A, MMP-1 activity was induced after treatment with various concentrations of Nac in a dose-dependent manner. In addition, there was a significant increase in MMP-1 level in the CCD-966SK cells after treatment with 1.0 mM Nac for 24 h.

We then examined the proteins involved in regulating the expression of MMP-1. Western blot analysis showed that Nac induced dose-dependent increases in PI3K levels by 12%, 31%, and 74% compared to those of the controls. The expressions of phospho-PKC (p-PKC), PKC, phospho-Jak (p-Jak1), Jak1, phospho-Stat 3 (p-Stat 3), Stat 3, *c-Fos*, and Jun proteins were also increased by 263%, 44%, 206%, 34%, 316%, 169%, 68%, and 64%, respectively, compared to the controls after treatment with 1.0 mM Nac for one day. Furthermore, stimulation with various concentrations of Nac was not associated with any significant changes in extracellular signal-regulated protein kinases 1 and 2 (Erk1/2) expression (Figure 5B). In addition, in order to clarify the effect of Nac post-wounding, the wounded cells were collected and assayed for the expression of PI3K, PKC, Jak, and Stat by Western blotting. The expressions of PI3K, p-PKC, PKC, p-Jak, Jak, p-Stat 3 and Stat 3 proteins were also increased by 202%, 266%, 35%, 188%, 76%, 282%, and 132%, respectively, compared to the controls after treatment with 1.0 mM Nac for one day (Figure 5C).

#### 2.1.6. Effect of Nac on Healing of Wound Closure

**Figure 6.**
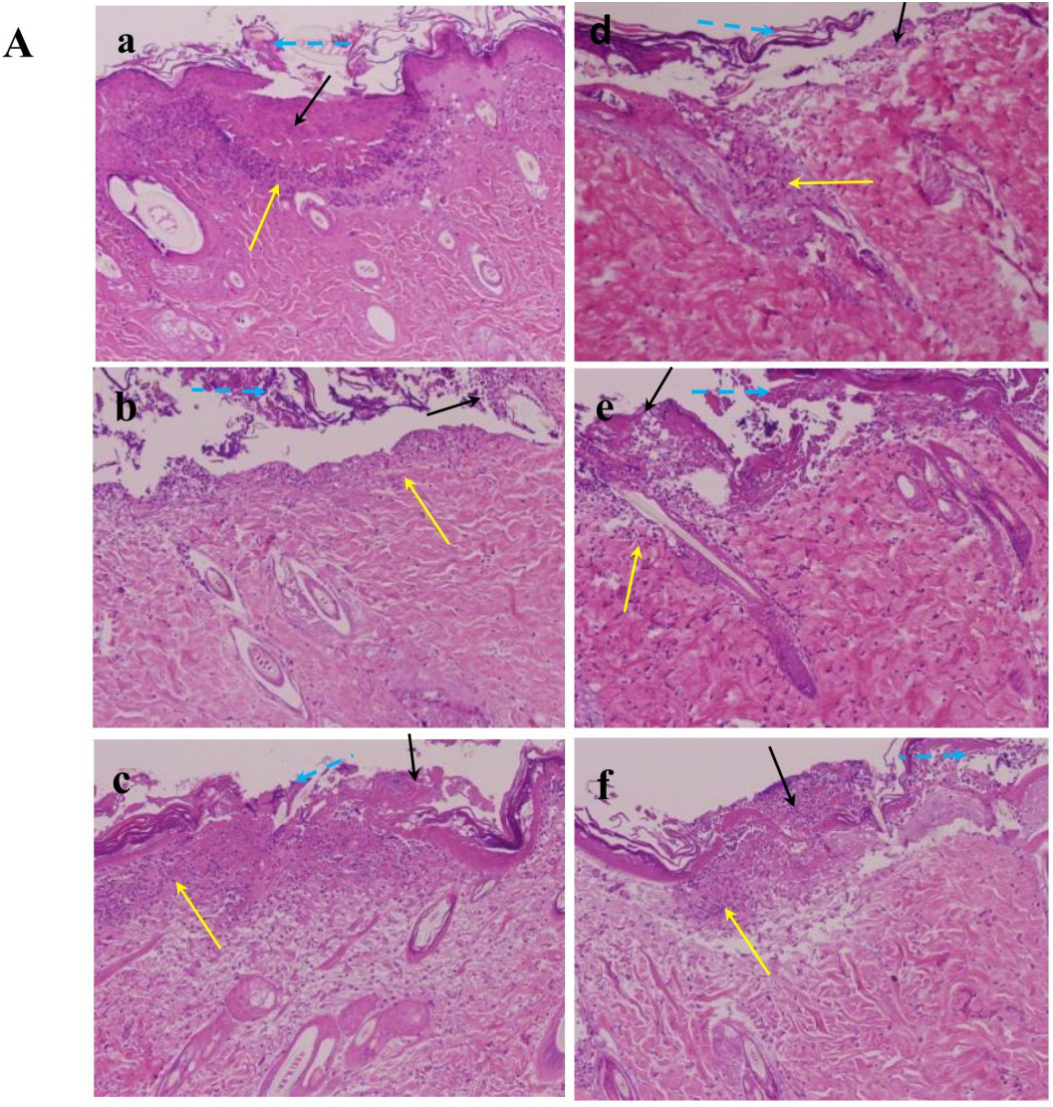

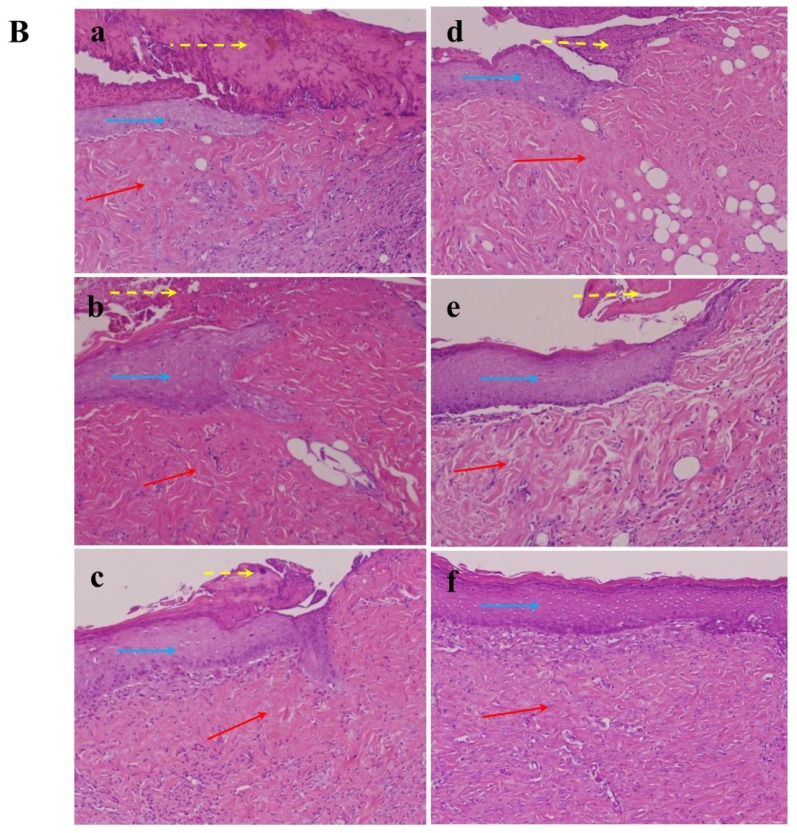
Histological features of the burn wounds on (**A**) day 3 and (**B**) day 13 after topical treatment with untreated controls (C) (**a**), silver sulfadiazine 1% cream (positive controls, PC) (**b**), Vaseline (vehicle) (**c**) and various concentrations of Nac (0.1%, 0.5%, 3.0%) (**d**–**f**). Yellow arrows indicate acute inflammation; black arrows indicate necrotic tissue; blue dashed arrows indicate peeling of epithelial tissue; red arrows indicate collagen formation; yellow dashed arrows indicate crust; blue arrows indicate regenerated epidermis. Photos shown are all representative of three independent experiments done in duplicate. The images in (**A**) and (**B**) show the original magnification 100×.

Three days after injury, typical healing responses of the inside epidermis of the wound were noted with topical treatment of 0.1%, 0.5%, 3.0% Nac, silver sulfadiazine cream (PC, positive control), and in untreated controls (C). We observed that the wounds were brown, dry and smaller in all animals whether or not they were treated with reagents. Similarly, rat punch biopsy tissues stained with hematoxylin and eosin were examined. Necrotic tissue, acute inflammation and peeling of epithelial tissue were seen in all wounds of all animals (Figure 6A).

In the wound healing process, a crust is formed by accumulating fluid, cells, and clotting materials in the early phase. Collagen and regenerated epidermis formation occurs in later wound healing stages. The reduction in crust thickness is one parameter of later wound healing [24]. The results indicated that the mortality and toxic reaction rates during the study were zero, and that the observed wounds were not significantly different with respect to wound closure among all groups on day 13. The histological findings at 13 days after injury of the C, PC, vehicle and conditioned Nac (0.1%, 0.5%, 3.0%) rats showed that, excluding 3.0% Nac-treated wounds, all wounds formed a crust and the largest crust was seen in the control group. Collagen and regenerated epidermis were displayed in all animals, however 3.0% Nac-treated wounds showed better integrality on re-epithelialization compared with the other groups. These results appear to demonstrate that 3.0% Nac exhibited a better ability to promote wound healing compared with the C, PC, vehicle, 0.1%, and 0.5% Nac rats (Figure 6B). We then quantified the regenerated epidermis of each group with Image Database Update to Image-Pro Plus (IPWIN60). The ratios of regenerated epidermis were calculated as percentage of the overall area of picture and all the pictures were with the same magnification of 100×. The percentage of each group was as follows (%): (a) untreated control, 8.60 ± 3.33; (b) positive control, 14.90 ± 2.56; (c) vaseline, 10.94 ± 3.19; (d) 0.1% Nac, 10.74 ± 1.47; (e) 0.5% Nac, 14.47 ± 0.80; (f) 3.0% Nac, 19.02 ± 2.85. Compared with untreated control wounds, the percentages of 0.5% and 3.0% Nac-treated wounds were significantly higher (*p* < 0.05 and *p* < 0.01, respectively). In addition, the percentage of the regenerated epidermis of the 3.0% Nac-treated wounds was also significantly higher than those of positive control and vaseline (both *p* < 0.05).

### 2.2. Discussion

Our results demonstrated that Nac significantly increased the concentration of GSH in CCD-966SK cells in a dose-dependent fashion. It has been shown that the production of ROS in cutaneous injuries inhibits the healing process by causing damage to cellular membranes, lipids, proteins, and DNA. High levels GSH will reduce the free radical damage and promote wound healing [25]. In our study, the GSH levels in CCD-966SK cells following treatment with 1.0 mM Nac were significantly increased compared with those of the controls, and histological studies of the rats’ wounds treated with 3% Nac revealed significantly better closure at day 13 after wounding compared with the controls or vehicle. These findings suggest that topical application of Nac may have promising beneficial effects with improved wound healing activities. In the literature, accumulating evidence shows that Nac promotes corneal wound healing, and the most commonly used dose of Nac in these experiments was 3% [26,27]. Thus, in the present study 3% Nac was selected for the experimental conditions.

The PKC family of molecules are important in the regulation of cellular differentiation, proliferation, apoptosis, adhesion and migration, and involve the classical upstream PKC regulator PI3K [28]. Acute wound repair involves several processes that are mediated by growth factors, cytokines, and other mediators released in the injured tissues or cells (keratinocytes, fibroblasts, and endothelial cells) [1]. Upon binding to their specific receptors, these cytokines activate numerous signaling pathways, including the PI3K/PKC family and transcription factors from the Stat family of proteins. In the present study, Nac remarkably enhanced the proliferation of CCD-966SK cells and increased the expression of PI3K, indicating that 3% Nac is capable of promoting wound healing activity.

Angiogenesis, the process of new blood vessel growth, is a key element in the proliferative phase of healing. Vascular endothelial growth factor (VEGF)-mediated Stat3 activation has been shown to be essential for migration and tube formation in human dermal microvascular endothelial cells [29]. Furthermore, Takeda and Akira observed that wound healing was markedly delayed when Stat3-mutant mice were wounded with a punch biopsy, indicating that Stat3 is essential for skin remodeling [30]. Our results revealed that Stat3 is one of the key targets of Nac as evidenced by the significantly increased expression of Stat3 following Nac stimulation.

Formation of the ECM is necessary for wound healing, and MMP-1 is required in the epidermis to facilitate re-epithelialization by remodeling the basement membrane, promoting cell elongation and actin cytoskeletal reorganization, and activating Erk signaling [31]. In our study, the expression of MMP-1 was significantly increased by Nac simulation, however the expression of Erk1/2 was not significantly different compared to the controls. These results support the notion that an increased expression of MMP-1 is likely to be induced in the PKC signaling pathway via Stat3.

## 3. Experimental Section

### 3.1. Ethics Statement

The experimental animals used in this study were handled according to the guidelines of the Institutional Animal Care and Use Committee of Chung Shan Medical University (IACUC, CSMU, Taichung, Taiwan), and the protocol was approved by IACUC, CSMU (Permit Number/Date: 925/29 June 2010). All surgery was performed under isoflurane inhalant anesthesia, and all efforts were made to minimize suffering.

### 3.2. Cell Culture and Preparation of Nac

CCD966SK, a human skin fibroblast cell line, was obtained from BCRC (Bioresource Collection and Research Center, Hsinchu, Taiwan). Cells were cultured in Dulbecco’s Modified Eagle’s Medium (DMEM) supplemented with 10% fetal calf serum, 2 mM l-glutamine, 100 U/mL penicillin and 100 mg/mL streptomycin mixed antibiotics, and 1 mM sodium pyruvate, purchased from Gibco/BRL (Gaithersburg, MD, USA). All cell cultures were maintained at 37 °C in a humidified atmosphere of 5% CO_2_–95% air. Nac was purchased from Sigma Corporation (Sigma 7250, St. Louis, MO, USA). A stock solution of 2.5 mM Nac was obtained by a 45 μm filter. Various concentrations of Nac (0.1, 0.5, 1.0 mM) were prepared by diluting the 2.5 mM stock with water.

### 3.3. Assessment of Cell Viability

MTT (3-(4,5-dimethylthiazol-2-yl)-2,5-diphenyltetrazolium bromide) assay was performed to determine cell viability. Cells were seeded at a density of 1 × 10^4^ cells/mL in a 24-well plate for 24 h. The cells were then treated with Nac at various concentrations (0, 0.1, 0.5, and 1.0 mM) for 1, 2, and 3 days. Afterwards, the medium was changed and incubated with MTT solution (5 mg/mL)/well for 4 h. After removing the medium, formazan was solubilized in isopropanol and measured spectrophotometrically (Hatachi 3210, Hitachi, Tokyo, Japan) at 563 nm.

### 3.4. Assessment of GSH

The concentration of GSH was determined by fluorescence spectroscopy, and a standard curve was established by given densities of GSH (0.04–4 mM). Cells were seeded at a density of 1 × 10^5^ cells/mL in a 24-well plate overnight. The cells were then treated with Nac at various concentrations (0, 0.1, 0.5, and 1.0 mM) for 3 days. A Cytofluor 2350 fluorescence plate reader (Millipore Corporation, Marlborough, MA, USA) was used to detect fluorescence using excitation and emission wavelengths of 350 and 420 nm, respectively. The data acquired were exported from the spectrophotometer using Cytofluor software.

### 3.5. Scratch Wound Healing Assay

CCD-996SK cells were seeded in 6-well plates and incubated overnight in DMEM with 10% fetal calf serum. After Nac treatment at various concentrations (0, 0.1, 0.5, and 1.0 mM) for 3 days, a confluent cell monolayer was wounded using a yellow P200 pipette tip, and then a cell-free area was created in each well. Given that cells respond by closing the wound through cell proliferation and migration, the migration of cells into the wound was monitored by capturing images at 0, 12, and 36 h. The healing ability of the CCD-996SK cells was quantified by counting the cells that had migrated into the scratch with a 400× objective in an Axioskop microscope (Zeiss, Olympus CK-2, Hamburg, Germany).

### 3.6. Boyden Chamber Migration Assay

A volume of 25 mg/50 mL Matrigel (Collaborative Biomedical Products, Bedford, MA, USA) was applied to polycarbonate membrane filters with 8 mm pores. Medium containing 10% fetal bovine serum (FBS) was applied to the lower chamber as a chemoattractant, and then Nac-treated cells were seeded on the upper chamber with 8-μm pore polycarbonate filters at a density of 1 × 10^5^ cells/well in 50 μL of serum-free medium. After incubating for 5 h, the cells that had migrated to the lower surface of the membrane were fixed with methanol and stained with 5% Giemsa solution, and the cells on the upper surface of the membrane were carefully removed with a cotton swab. The migrated cells were counted in five high-power fields (200×) using a light microscope. All samples were tested in triplicate, and the data are expressed as the mean ± SD.

### 3.7. Enzyme-Linked Immunosorbent (ELISA) Assay

The protein levels of MMP-1 in the media of the cells exposed to 0.1, 0.5, or 1.0 μM Nac were determined by ELISA (R&D Systems, Minneapolis, MN, USA). Test samples of 100 μL aliquots were added to 96-well plates for 24 h at 4 °C. The wells were blocked with bovine serum albumin and then incubated with the respective antibodies for 1 h at room temperature. The plates were washed with wash buffer, incubated with secondary antibodies linked to peroxidase for 1 hour at room temperature, washed, and subsequently incubated with peroxidase substrate until the development of color, which was measured spectrophotometrically at 450 nm.

### 3.8. Western Blotting Analysis

Whole-cell lysates (50 μg purified protein) were denatured and resolved on 10%–12% SDS-PAGE gels. After transferring the proteins onto nitrocellulose membranes (Millipore, Bedford, MA, USA), nonspecific binding of the membranes was blocked with Tris-buffered saline (TBS) containing 1% (*w*/*v*) nonfat dry milk and 0.1% (*v*/*v*) Tween-20 (TBST) for 2 h. The membranes were washed with TBST and incubated with specific primary antibodies overnight at 4 °C, washed with TBST and incubated with an appropriate secondary antibody (horseradish peroxidase-conjugated goat anti-mouse or anti-rabbit IgG) for 1 h. Band detection was revealed by enhanced chemiluminescence (ECL) using ECL Western blotting detection reagents and exposure to ECL hyperfilm on a FUJIFILM Las-3000 system (FUJFILM Co., Tokyo, Japan). The proteins were then quantitatively determined by densitometry using FUJFILM-Multi Gauge V2.2 software.

### 3.9. Wound Healing in Vivo Assay

#### 3.9.1. Preparation of Nac

Volumes of 0.1%, 0.5%, and 3% (*w/v*) Nac were mixed with Vaseline, and the reagents were stored in closed containers at an ambient temperature until use.

#### 3.9.2. Burn Wound Model

Thirty-six male Wistar rats (200 to 250 g) were used in the *in vivo* studies. The protocol used in this study was a modified version of the method described in a study by Nascimento *et al.* [32], and all rats were handled according to the guidelines of IACUC, CSMU. The animals were housed in individual cages with water and rodent chow provided ad libitum. The dorsum hair was shaved, and the rats were kept in their cages for 24 h before applying the burn protocol to minimize local inflammation. The experimental and control animals were given acetaminophen (1 mg/25 g) before burning. Each rat was exposed to isoflurane inhalant anesthesia, and the burn was then performed by holding a 1.0 cm^2^ steel square heated to 110 °C on the skin for 10 s at three locations on the rat’s back. Post-operative pain was treated with acetaminophen at a dose of 1 mg/25 g administrated to the rats three times a day for three days.

#### 3.9.3. Animal Treatment

The animals were divided into four groups, and three burn wounds were made on the back of each rat. The three wounds of each rat in Group I (*n* = 9) were respectively treated with 0.1% Nac, commercially available silver sulfadiazine 1% cream as positive control (PC), and no treatment as the control (C). The wounds of Group II rats (*n* = 9) were respectively treated with 0.5% Nac, PC, and C. The wounds of Group III rats (*n* = 9) were respectively treated with 3% Nac, PC, and C, and those of Group IV rats (*n* = 9) with 3.0% Nac, Vaseline as solvent control, and C. The wounds treated with Nac, PC, and Vaseline were subjected to the application of the reagents three times a day. The animals were observed daily, and 3 rats in each group were sacrificed at day 3, day 7, and day 13.

### 3.10. Histopathological Study

After the rats were sacrificed, full-thickness biopsies including adjacent skin, wound margin and epithelialized wound were taken and fixed in 4% formaldehyde, and then embedded in paraffin. Tissue slices were subjected to hematoxylin and eosin staining and histological studies by light microscopy. The slides were coded and examined a pathologist blinded to the study protocol to identify histological alterations.

### 3.11. Statistical Analysis

Data were expressed as mean ± SD of three independent experiments and were analyzed with one-way ANOVA and the Student’s *t*-test (Sigmaplot 11.0). Significant differences were established at *p* ≤ 0.05.

## 4. Conclusions

Nac has been widely used both orally and intraperitoneally in the treatment of various conditions, however the topical use of Nac to promote burn wound healing in rats has not been reported previously. In the present study, Nac showed remarkable activity related to cell proliferation, migration, scratch wound healing, and collagenous expression of MMP-1 via the PKC/Stat3 signaling pathway. Our results demonstrate that Nac can potentially promote wound healing activity, and may be a promising drug to accelerate burn wound healing.
